# Different Levels of Incomplete Terminal Pathway Inhibition by Eculizumab and the Clinical Response of PNH Patients

**DOI:** 10.3389/fimmu.2019.01639

**Published:** 2019-07-18

**Authors:** Markus J. Harder, Britta Höchsmann, Arthur Dopler, Markus Anliker, Christof Weinstock, Arne Skerra, Thomas Simmet, Hubert Schrezenmeier, Christoph Q. Schmidt

**Affiliations:** ^1^Institute of Pharmacology of Natural Products and Clinical Pharmacology, Ulm University, Ulm, Germany; ^2^Institute of Transfusion Medicine, University of Ulm, Ulm, Germany; ^3^Institute for Clinical Transfusion Medicine and Immunogenetics, German Red Cross Blood Transfusion Service Baden-Wurttemberg-Hessen and University Hospital, Ulm, Germany; ^4^Lehrstuhl für Biologische Chemie, Technische Universität München, Freising, Germany

**Keywords:** Eculizumab, paroxysmal nocturnal hemoglobinuria, PNH, complement, residual terminal pathway activity, Coversin, OmCI

## Abstract

**Background:** Eculizumab blocks the lytic complement pathway by inhibiting C5 and has become the standard of care for certain complement-mediated diseases. Previously, we have shown that strong complement activation *in vitro* overrides the C5 inhibition by Eculizumab, which accounts for residual terminal pathway activity.

**Results:** Here we show that the levels of residual hemolysis in *ex vivo* assays differ markedly (up to 3.4-fold) across sera collected from different paroxysmal nocturnal hemoglobinuria (PNH) patients on Eculizumab treatment. This large variability of residual activity was also found in sera of healthy donors, thus cross-validating the findings in patients. While PNH patients with residual lytic activities of 11–30% exhibited hemolysis levels around the upper limit of normal (i.e., plasma LDH of ~250 u/L), as expected for PNH patients on Eculizumab therapy, we found sustained and markedly increased LDH levels of around 400 u/L for the patient with the highest residual activity of 37%. Furthermore, the clinical history of nine out of 14 PNH patients showed intravascular breakthrough hemolysis at the time of documented infections despite ample amounts of administered Eculizumab and/or experimentally determined excess over C5.

**Conclusion:** The occurrence of extraordinary high levels of residual terminal pathway activity in PNH patients receiving Eculizumab is rare, but can impair the suppression of hemolysis. The commonly observed low levels of residual terminal pathway activity seen for most PNH patients can exacerbate during severe infections and, thus, can cause pharmacodynamic breakthrough hemolysis in PNH patients treated with Eculizumab.

## Introduction

Chronic intravascular hemolysis is the clinical hallmark of paroxysmal nocturnal hemoglobinuria (PNH) and occurs as a consequence of an un- or under-regulated complement alternative pathway (AP) (1, 2). Treatment with Eculizumab, a monoclonal antibody that targets the human complement component C5 and thus blocks the terminal and lytic complement pathway, has dramatically improved the clinical outcome and high mortality of PNH patients (3–8) [reviewed in Brodsky (9), Parker (10), Hill et al. (11), and Brodsky (12)]. Despite the remarkable clinical benefits under Eculizumab therapy, some serious issues remain. Some patients with a C5 single nucleotide polymorphism (SNP; p.Arg885His, rs56040400) fail to respond to Eculizumab altogether, while about one third of the patients do not respond completely and remain transfusion dependent (13–15). Different mechanisms can cause this incomplete clinical response, for example marrow failure or switch to extravascular hemolysis, i.e., removal of C3-opsonised PNH erythrocytes via the reticuloendothelial system (C3-opsonisation can still occur under therapy since Eculizumab only blocks the terminal complement pathway) (14, 16–18). We have recently elucidated an additional mechanism: contrary to dogma, Eculizumab or other stoichiometric C5-inhibitors reduce but do not completely block terminal pathway (TP) activity even in conditions with a huge excess amount of the inhibitors (19). The more profoundly complement was activated the less effective was the C5-blockade in several *in vitro* experiments. Therefore, our aim was to study the levels of residual TP activity and their impact on the clinical response (i.e., LDH levels, transfusion requirement) in a larger cohort of PNH patients. Here we observe residual TP activity under therapy with Eculizumab and provide evidence for its correlation with impaired response to Eculizumab.

## Materials and Methods

### Human Blood Components

Sera from 14 PNH patients or healthy volunteers were used under approval by the Ethical Committee of Ulm University (ethics committee vote “188/16”). Samples were collected in serum collection tubes according to the manufacturer's instruction, aliquoted, frozen in liquid nitrogen and stored at −80°C. Standardized normal human serum (NHS) was obtained from CompTech (US).

### Proteins

Eculizumab (Soliris™) was obtained from remnants in infusion pipes. A recombinant version of the natural tick-derived C5-inhibitor OmCI, denominated Coversin, was expressed with an N-terminal His6-tag in E. coli from a codon-optimized gene (20). The minimized engineered version of the natural AP regulator FH, miniFH, was produced as published (21, 22). Purified C5 was acquired from CompTech (US).

### Hemolysis Assays

AP activity was detected with a rabbit red blood cell (rRBC) hemolysis assay as described before (19, 23).

### Determination of Free C5 Levels in Patient Serum and NHS/Patient Serum Mixtures by ELISA

Eculizumab (50 μl of a 2 μg/ml solution) was coated onto 96-well microtiter plates (MaxiSorp; Nunc) as described previously (19). After washing, the plate was blocked with a 1% solution of BSA in PBS. Then, wells were exposed to a 1:200 dilution of a serum sample (50 μl). The samples consisted of PNH patient sera alone or mixed with NHS in ratios as specified. After washing, the C5 bound to the coated Eculizumab was detected by sequentially applying two detection antibodies (with intermediary washing): a polyclonal goat anti-human C5 antibody (Quidel), followed by a HRP-coupled donkey-anti-goat antibody, followed by color development with ABTS (Roche) in the presence of 0.03% H_2_O_2_. Absorbance was quantified at 405 nm.

## Results

**Figure 1:**
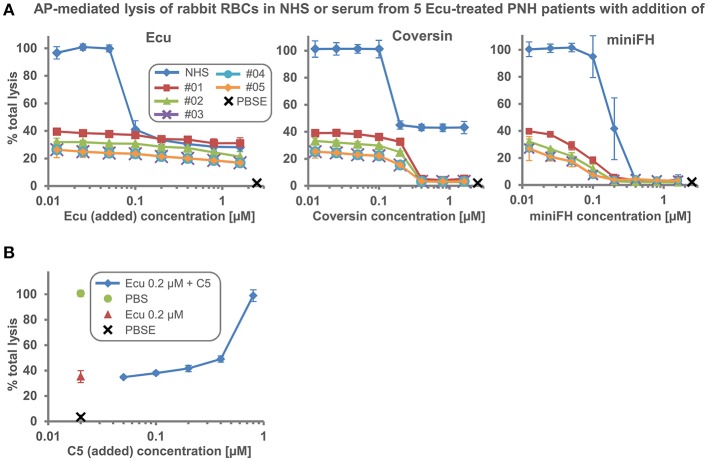
Inhibition of hemolysis by different complement inhibitors when added to NHS or five sera from PNH patients receiving Eculizumab treatment. **(A)** Alternative pathway (AP) mediated lysis of rabbit erythrocytes. Rabbit erythrocytes were incubated in 25% human serum in the presence of inhibitors or controls. NHS or serum from five patients receiving Eculizumab treatment was used (Average of 2 independent assays with SD is shown; data of patient sample #03 and #04 in this plot have been previously published in REF 19). **(B)** AP mediated hemolysis assay as in **(A)**. NHS was supplemented with 0.2 μM Eculizumab and increasing amounts of purified C5 were added into the reaction. Ecu, Eculizumab, NHS, normal human serum; PBSE, phosphate buffered saline supplemented with 5 mM EDTA; #01–05, numbering of PNH patients enrolled in the study.

**Figure 2:**
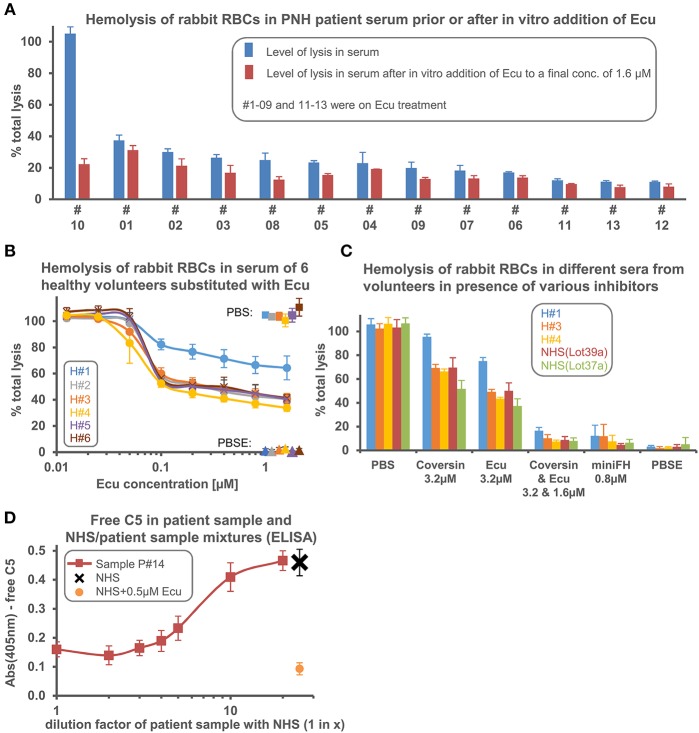
Serum from different individuals exhibits different levels of residual terminal complement pathway activity in the presence of excess amounts of Eculizumab or Coversin. **(A)** Residual hemolysis was tested in sera of 13 PNH patients as in Figure 1A, but this time only one concentration of Eculizumab was added. Hemolysis was measured in serum or in serum that had been supplemented *in vitro* with Eculizumab to reach a final concentration of “*in vitro* added Eculizumab” of 1.6 μM (final serum content was 25%). #01–09 and #11–13 were on Eculizumab treatment; thus, serum from these patients already contained Eculizumab prior to the *in vitro* addition of extra Eculizumab. Patient #10 was not on Eculizumab treatment. **(B,C)** Residual lytic activity in the presence of Eculizumab or Coversin was tested in sera from six healthy volunteers and in commercially available pooled NHS sources (the two different lots mean independent pools) which are certified to possess full complement activities (average of 3 independent assays with SD is shown). **(D)** Determination of free C5 levels. In a sandwich ELISA, Eculizumab was used as a capture antibody and a polyclonal anti-C5 as detection antibody. Eculizumab adsorbed to the microtiter plate captured free C5 from NHS, but not when NHS was pre-mixed with 0.5 μM Eculizumab. The addition of 0.5 μM Eculizumab to NHS served as a reference since the C5 concentration in NHS is about 0.5 μM. No free C5 was captured from the patient sample or patient samples that had been diluted 1:1 (1-in-2) with NHS (average of 3 independent assays with SD shown). H#1–H#6, numbering of healthy volunteers enrolled in the study. Other abbreviations see legend to Figure 1.

We employed a rabbit erythrocyte lysis assay with human serum that strongly activates complement to probe if sera obtained from different PNH patients on Eculizumab treatment exhibit variable levels of residual hemolytic activity. Because rabbit RBCs activate the AP so effectively, substantial levels of hemolysis can be observed even when Eculizumab is added into serum in excess of the C5 concentration (19). We found about three different levels of residual hemolysis in five patient sera (Figure 1A). Further increase of the Eculizumab concentration (by supplementing the patient serum *in vitro*) did not fully inhibit lysis. However, as described before, addition of the orthogonal C5 inhibitor Coversin (also known as OmCI, which binds to C5 on a site opposite to the epitope of Eculizumab) or the engineered alternative pathway (AP) inhibitor miniFH (which targets C3b and AP convertases) to the patient sera led to C5 double inhibition or proximal complement pathway inhibition, respectively, and thus resulted in complete inhibition of C5 activity (Figure 1A) (19). The fact that residual hemolytic activity in presence of Eculizumab can be suppressed by adding a different stoichiometric C5 inhibitor argues that residual activity in presence of only one C5 inhibitor is caused by incomplete C5 inhibition, and hence may be termed residual C5 activity. Titrating defined amounts of purified C5 into serum supplemented with 0.2 μM Eculizumab showed only modest increases in residual hemolytic activity until the point is reached when added C5 exceeds the neutralization capacity of Eculizumab, thus generating a steep increase in lysis (Figure 1B). Single point analyses of serum samples from 13 different PNH patients showed that the level of residual TP activity in this *ex vivo* assay differed markedly by a factor of up to 3.4 (Figure 2A). We also found a large variability of residual activities in sera from six healthy donors (about twofold), hence cross-validating that such variations in residual lytic activity are a more general phenomenon (Figure 2B). Intriguingly, after reaching the neutralization point of C5 by Eculizumab, which marks the expected steep decline in lysis, further addition of the inhibitor led to continuous but marginal further reductions in C5 activity. Sera of the healthy donors exhibiting different levels of residual TP activity were also probed with Coversin and showed the same ranking, albeit at higher residual activities (Figure 2C). As expected, double inhibition with Eculizumab and Coversin, or single inhibition with the AP inhibitor miniFH, stopped complement mediated lysis similarly to control levels. We then determined if an excess of Eculizumab over C5 can be detected in a sample from a patient (#14) who experienced a hemolytic crisis during a documented infection. NHS and NHS supplemented with 0.5 μM Eculizumab matching the C5 concentration in serum served as controls. The hemolytic patient sample was assayed in undiluted state and when it had been diluted in different ratios with NHS (Figure 2D). The ELISA signal as a measurement of free C5 only rose above background when the patient sample was mixed at least 1-in-2 with NHS indicating that lysis of erythrocytes had occurred in the patient despite an excess of Eculizumab over C5. Overall, these data demonstrate that the levels of residual TP activity in the presence of excess amounts of stoichiometric C5 inhibitors (i.e., Eculizumab or Coversin) vary across different individuals.

**Figure 3:**
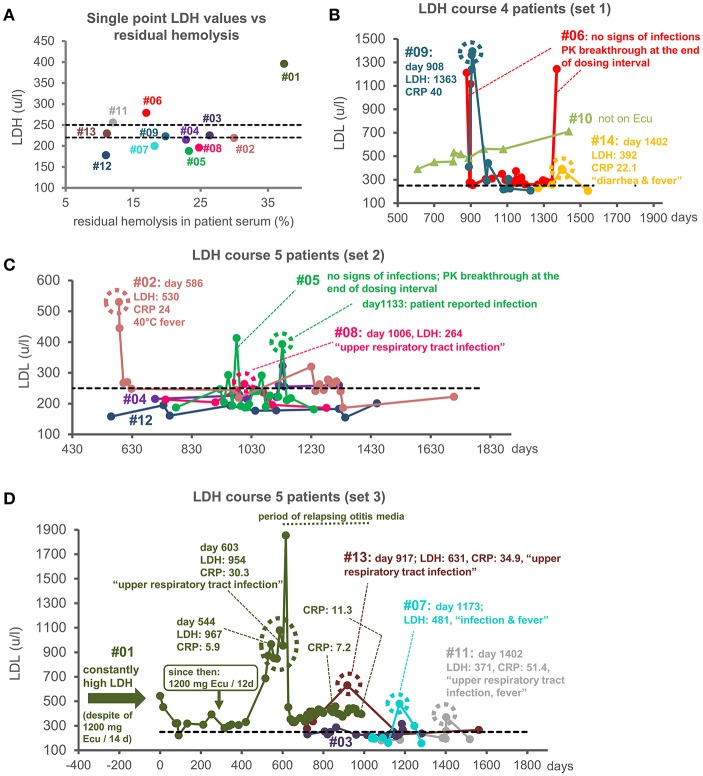
LDH as a measure of intravascular hemolysis. **(A)** LDH values (u/l) vs. residual hemolysis of patient sera. LDH values were measured from patients' samples at or close to the time of collection of serum, which was used for the determination of residual terminal pathway activity (see Figure 1). Only values from patients on Eculizumab treatment are shown. Two dotted black lines indicate the two existing reference values for the upper limits of normal. Two different LDH determination assays were used for the patients' samples: one with an upper limit of normal of 250 u/l and one with an upper limit of normal of 220 u/l. **(B–D)** LDH course of 14 PNH patients in subsets of four or five patients each. A dashed black line indicates the upper limit of normal for LDH of 250 u/l. The LDH values of the 13 patients that were on Eculizumab therapy are indicated by filled circles connected by a line. The LDH values for the patients not on Eculizumab therapy are indicated by triangles. For patient #06 **(B)** and, once, for #05 **(C)** high LDH values were measured at the end of the dosing interval with no concomitant signs of infections at these time points. For patient #01 constantly high LDH values were measured, which exacerbated during signs of severe infection **(D)**. A dashed circle indicates a LDH peak value at a time point for which signs of infections were documented. PK breakthrough stands for pharmacokinetic breakthrough (Units: LHD u/l; CRP: mg/l).

Next, we correlated the percentage of residual TP activity measured in patient sera with the LDH level at/around the time point of serum collection with LDH levels as surrogate for *in vivo* hemolytic activity in patients. Only the patient sample #01 with the highest residual TP activity of about 37% showed a substantially increased LDH level in this plot (Figure 3A). For all other patients, the LDH levels were around the upper limit of normal, as expected for PNH patients on Eculizumab therapy; for these latter samples (with residual hemolysis levels ranging from 11 to 30%) no correlation with LDH levels was evident. We then investigated the clinical time course of LDH levels in our cohort of PNH patients (Figures 3B–D). Only two patients showed substantially elevated LDH levels over an extended period: the only patient in the cohort not on Eculizumab treatment and patient #01 (Figures 3B,D). Irrespective of ample amounts of Eculizumab administered to patient #01 (1200 mg every 12 days; last Eculizumab application 9 days before serum sampling; see Supplemental Table 1) LDH levels remained elevated, resulting in transfusion-dependency. This shows that the rare occurrence of residual TP activity above 30%, as measured in our sensitive *ex vivo* assay, can be predictive for an incomplete clinical response.

Finally, we investigated the patient history for the samples that showed noticeable LDH peaks above the individual LDH course of a total of 14 patients (Figures 3B–D). For patient #06 (and once for #05) LDH peaks occurred at the end of the Eculizumab dosing interval and did not correlate with signs of infections, implying that the Eculizumab concentration fell below therapeutic levels; hence, these are interpreted as pharmacokinetic breakthroughs: i.e., reappearance of hemolysis toward the end of the dosing interval when the C5 concentration exceeds the one of Eculizumab (Figures 3B,C). Contrary to this situation, the marked but sporadic steep increases in LDH levels observed for nine patients correlated with signs of infections irrespective of the dosing interval. This argues that strong complement activation induced by pathogens may lead to a failure in completely blocking TP activity with Eculizumab (Figures 3B–D, Supplemental Table 2). Thus, clinical manifestations can also occur for patients with the commonly observed low to moderate levels of residual terminal pathway activity under Eculizumab: in cases of infections that strongly activate complement (24) the residual TP activity worsens and causes lysis of PNH erythrocytes alongside clinical symptoms. Supplemental Table 2 shows that hemolytic events frequently coincide with records of infections despite appropriate dosing of Eculizumab. This indicates that hemolysis during infections was not caused by low Eculizumab concentrations in circulation but by exacerbated residual TP activity. Indeed, in the hemolytic sample of patient #14 we could experimentally verify an excess of Eculizumab over C5 (Figure 2D).

## Discussion

Sera from two individuals, patient #01 and healthy volunteer #H1 (cf. Figure 2B), exhibited unusually high levels of residual TP activity in the presence of excess amounts of Eculizumab when the AP was activated strongly by adding rabbit erythrocytes to the Eculizumab supplemented sera. With regard to the cause of this higher residual hemolytic activity in patient #01 or in the healthy volunteer H#1, we speculate that the molecular reason may be a SNP in C5 or another relevant complement protein (e.g., a component that constitutes the C5 convertase). The sample size of 13 PNH patients and seven healthy volunteers does not allow to draw conclusions on the frequency of such occurrence of high residual TP activity, but nonetheless it suggests that such events are not particularly rare. The clinical implications are that patients with extraordinary high residual activity under C5 therapy benefit to a lesser extent from anti-C5 therapy regarding clinical symptoms and transfusion dependency. Importantly, these patients are not expected to benefit substantially from increasing the therapeutic dose above the level of C5 neutralization. Given that we observed extraordinary high residual lytic activity when the serum of H#1 was supplemented with Eculizumab or Coversin, we speculate that this high residual TP activity would also be observed for other stoichiometric C5 inhibitors. It would be of interest to also test the serum of patient #1 with regard to residual TP activity in the exclusive presence of Coversin, however, due to therapy with Eculizumab we could not obtain uninhibited serum of patient #1.

In contrast to the unusually high residual hemolytic activity of patient #1 and H#1, the sera of the other five healthy volunteers and 13 PNH patients exhibited comparatively moderate to low levels of residual lysis when challenged with the strongly AP activating rabbit erythrocytes. As shown here (for the majority of PNH patients) and before, substantial residual TP activity under Eculizumab therapy can only be detected *ex vivo* upon strong activation of complement pathways (19). However, the fact that the LDH levels of a big proportion of PNH patients do not completely normalize supports the notion that low levels of intravascular hemolysis (potentially of aged and thus more vulnerable PNH erythrocytes) may occur *in vivo* due to residual C5 activity in the presence of stoichiometric C5 inhibitors. Such residual C5 activity under Eculizumab therapy may hold the benefit of a continued immune surveillance against infections by a strongly dampened, but not completely inhibited TP. However, during severe infections that are followed by strong complement activation, which produces high C3b densities, the commonly observed low levels of residual TP activity in PNH patients exacerbate and thus causes pharmacodynamic breakthrough hemolysis in Eculizumab (or potentially other anti-C5) treated PNH patient. Hence, in cases of intravascular hemolysis due to infections (in spite of sufficient Eculizumab supply) the underlying principle is exacerbated residual TP activity, which is the mechanistic explanation of the term “pharmacodynamic breakthrough” that has been coined before (25, 26).

In conclusion, we show that extraordinary high levels of residual TP activity in certain individuals impair the suppression of hemolysis by Eculizumab and that residual TP activity exacerbates during infections, causing breakthrough hemolysis in Eculizumab treated PNH patients.

## Ethics Statement

Acquisition of peripheral blood material from patients and healthy volunteers enrolled in the present study as well as experiments performed with this material were in accordance with the recommendations of the Ethics Committee of Ulm University. All participants have signed the informed consent in accordance with the Declaration of Helsinki. The present study was approved by the Ethics Committee of Ulm University.

## Author Contributions

CS, MA, BH, and HS conceived the study. CS, AD, and MH performed the experiments. HS, MA, CW, TS, and BH advised on experimental design and data analysis. AS, CS, and MH provided and/or prepared essential protein reagents. The manuscript was written by CS, MH, and HS. All authors critically revised the manuscript and contributed to the discussion of the data.

### Conflict of Interest Statement

CS is an inventor of a patent application that describes the use of miniFH for therapeutic applications. BH, CS, and HS (to University of Ulm) received honoraria for speaking at symposia organized by Alexion Pharmaceuticals. HS and BH served on an advisory committee for and received research funding from Alexion Pharmaceuticals (to University of Ulm). HS served on an advisory committee for Ra Pharmaceuticals and Alnylam. The remaining authors declare that the research was conducted in the absence of any commercial or financial relationships that could be construed as a potential conflict of interest.
