# Supplementary material for: Different Levels of Incomplete Terminal Pathway Inhibition by Eculizumab and the Clinical Response of PNH Patients
Source: Front Immunol. 2019 Jul 18;10:1639. doi: 10.3389/fimmu.2019.01639 (PMC6657537; doi:10.3389/fimmu.2019.01639)
Supplement: Supplementary file 1 [file Presentation_1.pdf]

Supplementary Material

Different levels of incomplete terminal pathway inhibition by Eculizumab and the clinical response of PNH patients

Markus J. Harder, Britta Höchsmann, Arthur Dopler, Markus Anliker, Christof Weinstock, Arne Skerra, Thomas Simmet, Hubert Schrezenmeier,* Christoph Q. Schmidt*

*** Correspondence:**
Christoph Q. Schmidt: christoph.schmidt@uni-ulm.de
Hubert Schrezenmeier: h.schrezenmeier@blutspende.de

Supplemental Tables 1 & 2 **(page 2-3)**

Supplemental Table 1

Supplemental Table 1. Patient data of corresponding PNH sample around time point of sampling for single point analysis (corresponding to data in Fig. 1B)

**Pat#**	**Level (%) of residual hemolysis** (in vitro rabbit RBCs assay)	**RBCs trans-fusions** (units)	**Sex**	**Weight/ Height** (kg/cm)	**Age** (y)	**Ecu dose*** (every 14 days or specified otherwise) (mg)	**Days since last Ecu dose***
#01	37	6-8 units per year	m	110 / 174	27	1200 (from 06/2013 every 12 days)	9
#02 (1)	30	-	m	97 / 178	43	1200	9
#03	17	-	m	108 / 204	43	1200	12
#04	23	~ every months if Hb<9^{X}	f	64 / 167	27	900	5
#05	23	-	m	81 / 177	35	900	7
#06	17	every month 2	m	90 / 176	74	900	2
#07	18	-	m	105 / 186	34	900 every 12/14 days	14
#08	25	-	m	90 / 179	30	900	not known
#09	13	-	m	100 / 177	33	not known	14
#10	105	-	f	62 / 179	32	no Ecu treatment	-
#11	12	-	f	60 /168	36	900	8
#12	11	-	f	95 / 179	48	900	7
#13	11	-	m	110 / 183	60	1200 every 12 days	2
#14 (plasma instead of serum was collected)	not determined	on average 2 every month	f	57 / 168	52	900	12

*** samples acquisition includes samples from outpatients with irregular visits to the clinic which can lead to a lack of background data for some columns for individual patients (marked with "not known")**

^{X} Transfusion schedule during pregnancy: serum sample for measuring residual hemolysis was taken during the 2nd trimester of pregnancy

SUPPLEMENTAL Table 2

Supplemental Table 2. Clinical data around hemolytic peak (corresponding to data in Fig. 2B-D)

**Pat#**	**day of hemolytic event (post day = 0)**	**LDH value measured (u/l)**	**CRP (mg/L)**<br><br>**REF < 5**	**Clinical symptoms**	**Ecu dose (mg) *** (every 14 days or specified otherwise)	**Days since last Ecu dose***
#01	**constantly high**	constantly around 400	-	continuous high transfusion requirement	1200<br>(every 12 days)	not applicable
#01	544	967	5.9	-	1200<br>(every 12 days)	not known
#01	603<br>(616)	954<br>(1855)	30.3<br>-	upper respiratory tract infection	1200<br>(every 12 days)	not known
#02	586	530	24	40°C fever	1200	not known
#05	980<br><br>1133	413<br><br>393	-	no signs of infection<br><br>patient reported infection	900<br><br>900	14<br><br>13
#06	898<br><br>1370	1116<br><br>1244	-	no signs of infection<br><br>no signs of infection	1200<br><br>900	13<br><br>14
#07	1173	481	-	infection and fever	900<br>(every 12 days)	not known
#8	1006	264	-	upper respiratory tract infection	900	10
#09	908	1363	40		900	9
#11	1402	371	51.4	upper respiratory tract infection and fever	900	not known
#13	917	631	34.9	upper respiratory tract infection	1200	1
#14	1402	392	22.1	infect, diarrhea and fever	900	12

*** samples acquisition includes samples from outpatients with irregular visits to the clinic which can lead to a lack of background data for some columns for individual patients (marked with “not known”)**
